# Abatacept to treat chronic intestinal pseudo-obstruction in five systemic sclerosis patients with a description of the index case

**DOI:** 10.1177/2397198318766819

**Published:** 2018-04-10

**Authors:** Barbara Vigone, Lorenzo Beretta

**Affiliations:** Scleroderma Unit, Referral Center for Systemic Autoimmune Diseases, Fondazione IRCCS Ca’ Granda Ospedale Maggiore Policlinico, Milano, Italy

**Keywords:** Systemic sclerosis, gastrointestinal, abatacept, treatment

## Abstract

Chronic intestinal pseudo-obstruction is a severe complication of systemic sclerosis. Inflammatory neuropathy and immunological alterations have a prominent role in the development of systemic sclerosis–related chronic intestinal pseudo-obstruction and immunomodulation might be beneficial in this context. An accidental observation of a patient with juvenile arthritis and a biopsy-proven diagnosis of autoimmune ganglionitis led us to experiment with a new approach to treat systemic sclerosis–related chronic intestinal pseudo-obstruction. In our arthritis patient, the severity and frequency of recurrent episodes of chronic intestinal pseudo-obstruction and aspiration pneumonia were reduced whenever steroids were used to treat arthritic flares, which dramatically improved with abatacept therapy. A systemic sclerosis patient presented typical chronic intestinal pseudo-obstruction features that were neither controlled by dietary interventions nor by prokinetics and were often complicated by acute episodes (5-year) requiring hospitalization. Increased food tolerance was observed whenever parenteral steroids were used during hospitalization. An adequate long-term control of symptoms was then obtained with the use of intramuscular methylprednisolone 20 mg/day; however, symptoms promptly recurred after tapering. Following this motivating example, immunomodulation with abatacept was started. Symptoms were then well controlled and steroids could be weaned off without further acute episodes of sub-occlusion. We postulate that inflammatory neuropathy resembling myenteric ganglionitis may be suspected in selected systemic sclerosis patients with chronic intestinal pseudo-obstruction features. Immunomodulation with drugs that act on T function and restore the regulatory/effector T cell balance may be beneficial in these subjects. The outcomes of four additional systemic sclerosis patients with severe and refractory symptoms of intestinal pseudo-obstruction successfully treated with abatacept are also presented.

## Introduction

Chronic intestinal pseudo-obstruction (CIPO) is a life-threatening syndrome characterized by signs and symptoms of intestinal obstruction without evidence of mechanical lesions of the intestinal lumen.^1^ This syndrome predominantly develops in children as an idiopathic disorder, yet it can secondarily be observed in a number of diseases, especially in adults.^1^ The etiology of CIPO is unknown and it can be viewed as a form of insufficiency of the intestinal pump that is unable to promote the transit through the gut, due either to a lack of coordination or to a reduction in propulsive forces. CIPO can be classified into neuropathic, mesenchymopathic, and myopathic, depending on the involvement of enteric neurones, interstitial cells of Cajal or smooth muscle cells, respectively; sometimes more involvements may coexist in one patient.^1,2^ Inflammatory neuropathy is the most common form of enteric neuropathy and is characterized by lymphocytic infiltrates surrounding the myenteric plexus (myenteric ganglionitis), composed of both T helper and T suppressor cells in a 1:1 ratio.^2^

Systemic sclerosis (SSc) is often complicated by gastrointestinal dysmotility problems with up to 90% of patients experiencing symptoms related to the involvement of the upper or lower enteric tract, including severe and refractory forms of CIPO.^3^ The pathophysiology of enteric involvement in SSc is poorly understood and only few studies have tried to elucidate its mechanisms.^2,4^ A four-stage process has been postulated to explain SSc-related enteropathy. In the first stage, there is a direct endothelial involvement as observed in the skin. In the second stage, an inflammatory neuropathy characterized by inflammatory infiltrates in close association with plexuses develops. In the third stage, muscle atrophy appears to eventually evolve into end-stage lesions with fibrosis (fourth stage). SSc-related enteric involvement is usually severe and resistant to therapy; prokinetic drugs and dietary modification may be effective in patients with mild to moderate symptoms,^5^ while the management of nonresponsive cases complicated by pseudo-obstruction is often challenging.^2^

Herein, we report the case of an SSc patient with CIPO refractory to prokinetic and supportive measures that was successfully treated with abatacept (ABA). This approach was motivated by a casual observation made in a patient with arthritis and a previous biopsy-proven diagnosis of autoimmune ganglionitis with recurrent episodes of pseudo-obstruction that responded to ABA. The immunological implications of our observation are discussed and the outcome of four other patients with severe SSc involvement is presented as well.

## Case description

### Case 1—the motivating case

The patient came to our attention at the end of 2012, when he was 16. He had a history of juvenile rheumatoid arthritis and a biopsy-proven diagnosis of autoimmune ganglionitis made when he was 3. Autoimmune ganglionitis was beneficially treated with parenteral nutrition, cisapride, erythromycin, high-dose steroids, and azathioprine (AZA); years later, prokinetics and erythromycin were gradually reduced and stopped with worsening of the patient’s gastroenteric symptoms.

When he was 9, juvenile rheumatoid arthritis developed; arthritis was not well controlled by steroids (mean daily dose of prednisone equal to 10 mg) and AZA (150 mg/day); attempts to substitute AZA with methotrexate or cyclosporin A were characterized by worsening of intestinal dysmotility symptoms despite the improvement of articular manifestations. AZA was first coupled with infliximab (not tolerated for anaphylactic reaction) and then with adalimumab (ADA) with incomplete control of joint symptoms. The patient presented once or twice a month arthritic flares treated with short courses of medium-dose steroids (prednisone up to 25 mg/day for 1 week then gradually tapered to 10 mg/day). A marked improvement of intestinal symptoms was noticed whenever steroids were increased.

When the patient referred to us, he had disabling joint pain with a reduced articular function despite the ongoing treatment with AZA 150 mg/day, ADA 40 mg/2 weeks, and prednisone 10 mg/day. The patient also reported daily symptoms of gastroesophageal reflux-disease (GERD), complicated every 6 weeks by aspiration pneumonia requiring systemic antibiotics. He also reported severe constipation with complete intestinal bowel movements no more than twice a week. Reintroduction of erythromycin and prokinetics yielded no satisfactory effects on gastroenteric symptoms. Due to the persistent joint pain and functional limitations, decision was made to switch ADA to another biologic drug and ABA 750 mg IV/month was started. Joint pain relieved after two intravenous cycles; meanwhile the patient noticed a marked improvement of gastroenteric symptoms with increased bowel motility, reduced bloating, and a consistent reduction of GERD episodes. In the following months, the patient continued to feel well, complete bowel movements regularized to 4–6/week, and he had no further episodes of aspiration pneumonia so that it was then possible to reduce AZA to 100 mg/day and prednisone to 5 mg/day.

### Case 2—SSc-related CIPO case

A 44-year-old man came to our attention in July 2011 with a 9-month-old diagnosis of limited cutaneous SSc (lcSSc) characterized by Raynaud’s phenomenon, sclerodactyly, pathological capillaroscopy, interstitial lung disease (ILD), and GERD symptoms with endoscopic finding of grade A esophagitis. The patient tested positive for antinuclear antibodies, while the search for specific autoantibodies, including anti-Topoisomerase I, anti-centromere, anti-RNA polymerase III, anti-fibrillarin, and anti-SSa, was negative.

In relation to the presence of a ventilatory restrictive defect with a forced vital capacity <70% of predicted values and high-resolution computed tomography (CT) signs of ILD, treatment with oral cyclophosphamide (CYC) was advised. At the end of August, immunosuppression was discontinued due to the occurrence of diarrhea, nausea, loss of appetite, and vomiting unresponsive to empirical therapy with rifaximine 200 mg tid (three times a day). At the beginning of September, his abdominal symptoms further worsened with severe constipation and bloating. Abdomen X-rays showed air fluid levels in the small bowel and thickening of the haustra; colonoscopy was not performed due to suboptimal preparation, double-contrast barium enema showed no signs of mechanical obstruction. The patient was treated with parenteral nutrition, prokinetics and subcutaneous octreotide with slow recovery. He was dismissed at the beginning of November with the following therapy: omeprazole 20 mg bid, prednisone 25 mg/day, erythromycin 600 mg bid, Nifedipine 20 mg bid, and acetylsalicylic acid 100 mg/day.

**Figure 1.**
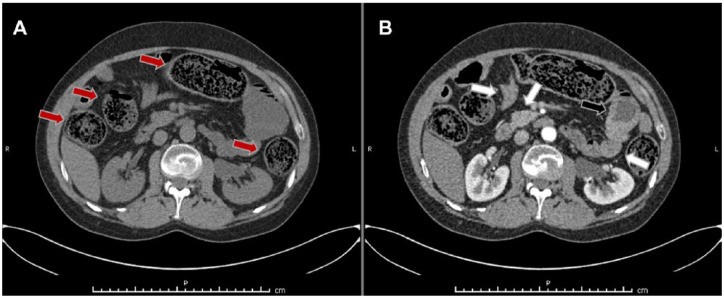
Abdomen computed tomography (CT) scan during the fifth episode of acute intestinal pseudo-obstruction. (a) and (b) CT scan images acquired before and after contrast administration. Abdominal computed tomography (CT) scan reveals diffuse distension of intestinal loops (red arrows) that dislocate and compress the liver. Diffuse hyperenhancement after contrast administration can be observed in the small intestine (white arrows) along with thickening (max 8–9 mm) of the intestinal wall (black arrow).

A few weeks after hospital discharge, an attempt to reintroduce oral CYC was made, yet the drug was permanently discontinued after an episode of pneumonia complicated by septic shock at the beginning of 2012. Concurrently, the patient experienced another (second) episode of intestinal pseudo-obstruction that resolved after nasogastric intubation and parenteral nutrition, which also required the (permanent) interruption of nifedipine and the temporary switch from oral prednisone to intravenous methylprednisolone (MTP). In March and June 2012, the patient experienced two further episodes of intestinal obstruction (third and fourth) successfully treated with nasogastric intubation and parenteral nutrition. A further episode of intestinal occlusion developed in November 2012 (fifth); at that time the patient had lost 17 kg (usual weight ≈ 75 kg). Oral food avoidance and parenteral nutrition were once again set and therapies were switched from the oral to the intravenous route. During hospitalization, the patient underwent a CT scan of the abdomen which showed a thickening of the cecum and of the intestinal walls (Figure 1). Colonoscopy showed no endoluminal lesions, a biopsy of the first tract of small bowel was performed showing a moderate lymphoplasmacellular infiltration in the lamina propria, mostly CD3+ cells.

Reviewing the patient’s case, it was observed that when all the sub-occlusive episodes resolved and the patient was still hospitalized, he could well tolerate increasing quantities of oral food. Yet, at home, without any change in the prescribed diet, intestinal symptoms gradually recurred. Apparently, the sole main difference was the switch from parenteral to oral steroids. At discharge from the last hospitalization, decision was then made to keep the patient on intramuscular (i.m.) MTP 20 mg/day. During the following months the patient felt well; however, whenever attempts were made to taper MTP, symptoms of incomplete intestinal pseudo-obstruction appeared.

Following the experience from the patient with juvenile rheumatoid arthritis and autoimmune ganglionitis, the decision was taken to introduce ABA 750 mg IV/month in April 2013. Bowel symptoms were soon controlled thereafter; steroid therapy was successfully switched to the oral route and then tapered till complete suspension 4 months later. In the following years the patient regained his usual weight and had no further episodes of sub-occlusion, even if he seldom experienced short periods of constipation and nausea that spontaneously receded.

### Case series of SSc-CIPO patients treated with ABA

Since the beginning of 2014, four additional SSc patients with intestinal involvement characterized by severe constipation, multiple episodes of CIPO requiring hospitalization, or early symptoms of incomplete intestinal pseudo-obstruction not requiring hospitalization (resembling König syndrome) were treated with ABA. All the patients were unresponsive or partially responsive to supportive measures. All the patients were first treated with i.m. MTP and then ABA to reach a long-term control of intestinal symptoms (Table 1). GERD symptoms were partially, yet incompletely relieved with treatment.

**Table 1.** Characteristics of ABA-treated patients.

	Case 1 (index)	Case 2	Case 3	Case 4	Case 5
Gender	M	F	F	F	F
Subset	lcSSc	lcSSc	lcSSc	lcSSc	dcSSc
Autoantibody	ANA	Negative	ACA	ACA	ANA
Age at disease onset (in years)	41	63	52	29	52
Disease onset (calendar year)	2011	2010	2003	1986	2007
ABA start (calendar year)	April 2013	January 2014	December 2015	January 2016	June 2016
Complete IPO					
Before ABA	5	5	3	2	0
After ABA	0	0	0	0	0
Incomplete IPO					
Before ABA	4	10	0	>100^a^	>100^b^
After ABA	0	1	1	2	5
CSBM					
Before ABA	<3/week	3–6/week	<3/week	<3/week	<3/week
After ABA	1/day	1/day	2/day	1/day	3/week
Weight (kg)					
Before ABA	58	37	49	45	44
After ABA	74	40	53	47	47
Albumin (g/dL)					
Before ABA	3.1	3.3	3.6	3.8	3.6
After ABA	3.7	3.3	3.9	3.7	3.7
Other therapies	Ery; rATB	Pru	Pru; rATB	Pru; rATB	Pru; rATB

ABA: abatacept; lcSSc: limited cutaneous systemic sclerosis; dcSSc: diffuse cutaneous systemic sclerosis; ANA: antinuclear antibodies; ACA: anti-centromere antibodies; Complete IPO: episodes of intestinal pseudo-obstruction requiring hospitalization; Incomplete IPO: incomplete episodes of intestinal pseudo-obstruction responsive to supportive measures and not requiring hospitalization; CSBM: complete spontaneous bowel movements; Ery: erythromycin; Pru: prucalopride; rATB: rotation antibiotics.

a At least 1/month.

b At least 2/month.

## Conclusion

The pathophysiology of SSc-related intestinal involvement is poorly understood, yet immunological aspects, as highlighted by the occurrence of alterations ranging from the presence of inflammatory infiltrates to the positivity to myenteric neuronal antibodies,^2,4^ seem to play a prominent role in the development of this complication. Visceral neuropathy with inflammatory features is a cardinal feature of enteric dysmotility syndromes and of CIPO.^6^ Therefore, tackling immune system activation may be beneficial in some patients with SSc and severe intestinal involvement.

In our patient, gut biopsy showed a moderate lymphoplasmacellular infiltration in the lamina propria, with predominance of CD3+ T cells. Although a full-thickness specimen of the intestinal wall—required to confirm the diagnosis of inflammatory neuropathy—was not available, it is noteworthy to observe that CD3+ infiltrate is prototypical of autoimmune ganglionitis. Steroids are the anchor therapy of autoimmune ganglionitis^1^ and a striking clinical response was observed when medium-dose steroids were administered in our patient, albeit the response was limited to parenteral MTP, most likely due to the effect of malabsorption on prednisone kinetic. Prednisone and MTP are potent inducers of regulatory T cells (Treg) while they reduce the activity and the number of effector T cells (Teff).^7^ Remarkably, a favorable effect of MTP on CIPO secondary to SSc has already been described by Ortiz-Alvarez et al.^8^ In our experience, a marked and sustained benefit was observed when ABA was introduced in our patients, reducing steroid dependence. ABA provided long-term efficacy in those who initially responded to parenteral steroid therapy that was used as an empirical proof for the presence of an inflammatory (autoimmune) dysmotility syndrome. It is noteworthy to underline that in our experience, response to MTP was the prerequisite to treat patients with ABA and that we never treated MTP-nonresponding subjects with ABA (data not shown).

The cytotoxic T lymphocyte-associated Ag-4 (CTLA-4) is highly expressed on Tregs, is decisive in their suppressive function, and the molecule promotes Treg differentiation and accumulation at the intestinal level. CTLA4-Ig acts by binding to CD80 and CD86 receptors on antigen-presenting cells (APCs) inhibiting T cell activation and proliferation, blocking the specific interaction of CD80/CD86 receptors to CD28. ABA was shown to promote Treg generation and function by inducing tolerogenic dendritic cells. Tregs rely on CD28-dependent signals for development and CTLA-4 blockade may have a negative effect on the number of circulating Tregs, although conflicting results have been described in the literature. Some authors describe a reduced proliferation and activity of Tregs after CD28 blockade, while others describe a preserved or an increased regulatory function following CTLA4-Ig administration.^9^ It has been hypothesized that ABA inhibits Tregs blocking the CD80/86-CD28 interaction and, on the other hand, it induces or expands Treg in the periphery via tolerogenic dendritic cells so that the balance between T effector cells and Treg-cell activation is switched in favor of Treg-cell activity. Thus, ABA may sustain, promote, and practically substitute the effect of steroids in ganglia, dampening the detrimental effect of Teff in favor of Treg immunomodulation. Of interest, high-dose intravenous immunoglobulins were shown to be effective in SSc patients with enteric dysmotility^10^ and this kind of treatment has been associated with an increase in Treg.^10^

Further efforts are needed to characterize the immunological implications of severe SSc-related intestinal involvement and the effect immunomodulation may have on this complication. Results from the ongoing study on ABA in SSc (ClinicalTrials.gov Identifier: NCT02161406) may provide some clues about these aspects even if more focused studies on selected patients with enteric dysmotility and/or CIPO are warranted.
